# Congenital Rubella Syndrome in The African Region - Data from Sentinel Surveillance

**Published:** 2018-08-02

**Authors:** Balcha Masresha, Messeret Shibeshi, Reinhard Kaiser, Richard Luce, Regis Katsande, Richard Mihigo

**Affiliations:** 1 WHO Regional Office for Africa. Brazzaville, Congo; 2 WHO Inter-country Support Team for East and Southern Africa. Harare, Zimbabwe; 3 WHO Inter-country Support Team for Central Africa. Libreville, Gabon; 4 Formerly with the WHO Inter-country Support Team for East and Southern Africa. Harare, Zimbabwe

**Keywords:** Congenital Rubella Syndrome, Sentinel Surveillance, African Region

## Abstract

**Introduction:**

Rubella is a mild febrile rash illness caused by the rubella virus. The most serious consequence of rubella is congenital rubella syndrome (CRS), which occurs if the primary rubella infection occurs during early pregnancy, with subsequent infection of the placenta and the developing fetus.

**Methods:**

WHO supported countries to set up sentinel surveillance for CRS using standard case definitions, protocols, and case classification scheme. This descriptive analysis summarises the data from 5 countries which have been regularly reporting.

**Results:**

A total of 383 suspected cases of CRS were notified from the 5 countries as of December 2016, of which 52 cases were laboratory confirmed and 67 were confirmed on clinical grounds.

The majority (43%) of confirmed CRS cases were in the age group 6 – 11 months. The most common major clinical manifestation (Group A) among the confirmed cases is congenital heart disease (72%) followed by cataracts (32%) and glaucoma (10%).

**Discussion and conclusions:**

The number of years of reporting from these sentinel sites is too short to describe trends in CRS occurrence across the years. However, the limited surveillance data has yielded comparable information with other developing countries prior to introduction of rubella vaccine. As more countries introduce rubella vaccine into their immunisation programs, there is a need to ensure that all rubella outbreaks are thoroughly investigated and documented, to expand sentinel surveillance for CRS in more countries in the Region, and to complement this with retrospective record reviews for CRS cases in selected countries.

## Introduction

Rubella is a mild febrile rash illness caused by the rubella virus. In childhood, the illness is characterized by a transient, erythematous rash, low grade fever, post-auricular and sub-occipital lymphadenopathy, sore throat, red eyes, headache, malaise and anorexia^1^, ^2^.

The most serious consequence of rubella is congenital rubella syndrome (CRS), which occurs if the primary rubella infection occurs during early pregnancy, with subsequent infection of the placenta and the developing fetus. The risk for congenital defects has been estimated at 90% for maternal infection before 11 weeks of gestation. Nerve deafness is the single most common finding among infants with CRS. Unilateral or bilateral cataracts are the most serious eye finding, occurring in about a third of infants. Patent ductus arteriosus is the most frequently reported cardiac defect^1^, ^3^.

Diagnosis of rubella or CRS requires laboratory confirmation. The detection of rubella specific IgM is the most widely available method for routine laboratory diagnosis. In CRS cases, IgM antibodies are sometimes found for up to 1 year after birth, and persistence of IgG antibodies beyond 6 months of age has been detected. In addition, rubella virus can be isolated from nasopharyngeal, blood, throat, urine, and cerebrospinal fluid specimens from rubella and CRS cases^4^, ^5^.

Rubella is endemic in countries in the African Region, with a large proportion of cases occurring in children less than 15 years of age 6–10. However, susceptibility to rubella is known to occur in adults as well. In Burkina Faso, a sero-survey among 341 pregnant women found an overall sero-positivity rate of 95.0% for rubella-specific IgG antibodies indicating a continuous transmission of endemic rubella virus in Burkina Faso, posing a threat to non-immune pregnant women^11^. Similar results have been documented from other studies as well^12^.

The WHO African region has set a goal to eliminate measles by 2020^13^. However, currently, there is no Regional goal for rubella/ CRS elimination. WHO recommends that countries should take the opportunity offered by accelerated measles control and elimination activities to introduce rubella vaccine, and incorporate rubella/ CRS control or elimination activities into their immunisation programs^13^,^14^,^15^. GAVI support is available to eligible countries to introduce rubella vaccine as per the WHO guidelines^16^. As of September 2017, only 20 of the 47 countries in the African Region have introduced rubella containing vaccine in their immunisation schedules^17^.

One of the reasons for the low uptake of rubella vaccine in the African Region is the lack of information on disease burden. The WHO African Region is currently supporting countries to generate some information on rubella incidence as part of the measles case based surveillance system. Epidemiological information and blood specimens are collected from persons with suspected measles cases and tested for measles-specific immunoglobulin M (IgM) antibody using standard enzyme-linked immunosorbent assay (ELISA). If results become negative for measles IgM, sera are subsequently tested for rubella-specific IgM antibody using standard ELISA testing^5^, ^18^.

In the African Region, the number of CRS cases reported through the annual aggregate reporting system to the WHO comes to a total of 242 cases in the 10 years period between 2007 – 2016, ranging from 0 cases reported in the entire Region in 2008 and 2011 to 78 cases in 2015. During these years, no more than 4 countries reported at least 1 case of CRS per year, indicating the huge gaps in the reporting system^19^.

The incidence of CRS is underestimated in surveillance systems because of the fact that CRS is not a common condition, the subtle nature of some of the clinical manifestations, the technical difficulty in diagnosing the condition, the lack of an organized surveillance system, and more importantly the lack of facilities to confirm the cases by laboratory means. In the African Region, it is estimated that 38,712 cases occurred in 2010, while the global estimate was 105,391^20^. In 2013, a study estimated the incidence of CRS to be 69/100,000 live births in DRC, corresponding to 2,886 infants (95% CI 342, 6395) born with CRS per year^21^.

WHO supported 11 countries to set up CRS sentinel surveillance between 2011 and 2016 in order to better document the occurrence of CRS so as to help in the initial decision to introduce rubella vaccine and also to help in monitoring impact following introduction. The sentinel surveillance sites from 5 of these countries (Zimbabwe, Tanzania, Rwanda, Burkina Faso, Zambia) have been regularly reporting to the WHO. Burkina Faso, Zimbabwe and Tanzania introduced rubella vaccine in their routine immunisation program following catch-up supplementary immunization activities in 2015, while Rwanda and Zambia introduced it in 2013 and 2016 respectively.

## Methods

Countries identified sentinel surveillance sites, especially tertiary care and specialty centers, based on the presence of clinical expertise and the likelihood of seeing infants with suspected CRS. Following the detection of a suspected case in the sentinel sites, surveillance focal persons identified from among the clinicians on the site examine the child and collect relevant demographic and clinical information using a standardised case investigation form. Serum specimen is collected for laboratory testing for rubella IgM, and laboratory testing is done in the accredited national measles and rubella serological laboratory using standard ELISA testing as per the existing protocols^4^, ^5^. WHO AFRO standard case definitions and algorithm is utilised for the classification of CRS cases^18^, ^22^.

A suspected case of CRS is any infant less than one year of age in whom a health worker suspects CRS, either from maternal history of suspected or confirmed rubella during pregnancy, or when the infant presents with one or more of the following: heart disease; suspicion of deafness; eye signs including any of these- cataract, diminished vision, nystagmus, squint, micropthalmos, congenital glaucoma.

A clinically-confirmed case is one in which a qualified physician detects in the infant

- two major clinical manifestations (group A): OR
- one clinical manifestation from group A and one of the minor manifestations from group B:

Group A clinical signs include: *Cataract(s), congenital glaucoma; congenital heart disease; hearing impairment; pigmentary retinopathy.* Group B clinical signs include: *Purpura; splenomegaly; microcephaly; mental retardation; meningo-encephalitis; radiolucent bone disease; jaundice with onset within 24 hours of birth*.

A laboratory-confirmed CRS case is an infant with a positive blood test for rubella IgM who has clinically-confirmed CRS. An infant with a positive blood test for rubella IgM who does not have clinically confirmed CRS is classified as having congenital rubella infection^18^.

The surveillance and laboratory information which is captured using a standard case investigation form is later entered into a computerized database at each sentinel site, and is shared with WHO on a monthly basis. We reviewed the sentinel surveillance data submitted from the 4 countries (Burkina Faso, Rwanda, Zimbabwe, Zambia, Tanzania) that had regularly shared data with the WHO. We used descriptive analysis to characterize the demographic and clinical characteristics of the cases.

## Results

A total of 383 suspected cases of CRS were notified from the 5 countries as of December 2016. Tanzania reported 152 suspected cases while 143 were reported from Zimbabwe. Fifty five percent of the suspected cases (211 out of 383) were classified as discarded (not CRS cases) as per the case classification algorithm, while 52 were laboratory confirmed and 67 were confirmed on clinical grounds. (Table 1)

The clinical picture of the suspected cases indicates that 51% were reported with a congenital heart disease, while 22 had cataracts. Hearing impairment was reported in only 5% of the suspected cases. Among the group B clinical signs, microcephaly was reported in 32% and neonatal jaundice in 30%. (Table 2). A history of maternal febrile rash during pregnancy was elicited in only 41 (10.7%) of the suspected cases.

Out of the 119 confirmed CRS cases, 65 were from Tanzania and 48 were reported from Zimbabwe, while Burkina Faso and Rwanda did not have any confirmed case. (Table 3) Burkina Faso did not classify 41 of the reported 69 cases, and these make up the majority of the 53 cases that were not classified at all in the databases from the respective sentinel surveillance sites. (Table 1) The majority (43%) of confirmed CRS cases were in the age group 6 – 11 months, while 21 were newborns. (Table 4)

The most common major clinical manifestation (Group A) among the confirmed cases is congenital heart disease (72%) followed by cataracts (32%) and glaucoma (10%). Among the Group B manifestations, microcephaly and splenomegaly were detected in 34% and 25% of the confirmed cases respectively. (Table 5)

## Discussion

Zimbabwe established sentinel CRS surveillance in 2011 while Zambia and Tanzania started implementing it in 2014, and Rwanda and Burkina Faso in 2015. The number of years of reporting from these sentinel sites is too short to describe trends in CRS occurrence across the years. However, the limited surveillance data has yielded comparable information with other developing countries prior to introduction of rubella vaccine.

In this series, hearing impairment was detected in only 5% of the suspected cases. This is an indication of the difficulty to diagnose the condition in early infancy. Half of the suspected cases (51%) and 72% of the confirmed cases presented with congenital heath disease, since the sentinel sites were mostly cardiac disease follow-up clinics in tertiary care centers. Cataracts were detected in 22% of the suspected cases and in a third of the confirmed CRS cases in this series. In the Sudan, among 31 suspected CRS cases under 1 year of age, 11 cases were serologically confirmed, with cataracts (81.8%) and cardiac disease (72.7%) being the most common clinical signs^23^.

In countries implementing rubella and CRS elimination activities, WHO recommends to establish febrile rash illness surveillance integrated with the surveillance for measles, along with the investigation of rubella outbreaks. In addition, WHO recommends the conduct of active surveillance for CRS following rubella outbreaks to detect and confirm suspected cases in the infant cohorts^22^, ^24^. In the case of these 5 countries in our study, the timing and location of the sentinel sites did not include considerations to the presence of rubella epidemics. In Vietnam, CRS was diagnosed in 292 infants following a rubella epidemic, and the most common clinical symptoms were congenital heart disease (91.4%), cataract(s) (45.2%), splenomegaly (38.7%) and purpura (32.9%)^25^. In the Solomon Islands, following a rubella outbreak in May 2012, prospective CRS surveillance done in various clinical settings over a 4 months period in 2013 and helped to identify 13 CRS cases^26^.

The CRS sentinel surveillance protocol indicates that the target age group for prospective CRS is infants less than one year of age because of the reliance on IgM testing, and the eventual decline in IgM levels. Although IgM antibodies may persist for up to 1 year, the rate of positivity declines after 6 months of life. Ideally, laboratory confirmation of CRS in an infant aged over six months should not rely on the IgM test alone if the result is negative. In such cases, serial IgG testing should also be included to check for a sustained level of antibody over several months^18^, ^22^. In this series, children older than 12 months were included in the reporting from Zimbabwe, and there were cases with missing information on age, indicating the need to ensure that the protocols are strictly followed.

There is also the need to document the viral strains of detected rubella viruses. Molecular epidemiology that allows the differentiation of circulating rubella viruses, can be used to monitor the transmission pathways and to identify interruption of endemic virus transmission. There is a gap in the African Region with regards to the baseline data on circulating viruses^27^. As of 2015, only Algeria and DR Congo had documented rubella genotype data from among the 47 countries in the Region^28^. Unfortunately, none of the 5 countries reporting from the sentinel surveillance sites had documented the strains of rubella viruses from the lab confirmed CRS cases.

## Conclusion and recommendations

In conclusion, the sentinel surveillance sites have managed to document suspected and confirmed cases of CRS within a short period of time, indicating that sensitive sentinel surveillance systems can play a role in the decision to introduce rubella vaccine and monitor the impact thereafter. As more and more countries prepare to introduce rubella vaccine into their immunisation programs, there is a need to ensure that all rubella outbreaks are thoroughly investigated and documented, to expand sentinel surveillance for CRS in more countries in the Region, to institute a mechanism to assure high sensitivity of case detection, and to complement this with retrospective record reviews for CRS cases in selected countries.

## Limitations

This analysis does not take into account the rubella incidence in the countries at the time. It covers only 5 countries, and sentinel surveillance was operational for no more than 3 years except in the case of Zimbabwe. Therefore the findings may not be representative of the overall trends in the countries, and of the epidemiological situation in other countries in the subregion. The identification of the sentinel surveillance sites was done at country level, depending on the presence of paediatric tertiary care and specialty centers in each country, and the availability of existing working arrangements with the institutions. Even though the case definitions and case investigation were standardised, currently there is no mechanism to assure that all suspected cases are captured through the sentinel surveillance system, with the possibility that milder cases may have escaped detection.

## Figures and Tables

**Table 1:** Classification of suspected cases of CRS by country. 2012 – 2016.

	Clinically confirmed CRS	Lab confirmed CRS	Congenital Rubella Infection	Discarded	Not classification	TOTAL
Burkina Faso	0	0	0	28	41	69
Rwanda	0	0	0	5	0	5
Tanzania	48	17	0	83	4	152
Zambia	3	3	0	0	8	14
Zimbabwe	16	32	0	95	0	143
TOTAL	67	52	0	211	53	383

**Table 2:** Clinical signs in 383 suspected CRS cases. 2012 – 2016. Sentinel sites in 5 countries in AFR.

Clinical sign		Number of cases	% cases
Group A	Congenital Heart Disease	195	51%
	Cataracts	83	22%
	Pigmentary retinopathy	26	7%
	Glaucoma	22	6%
	Hearing impairment	20	5%
Group B	Microcephaly	121	32%
	Jaundice	113	30%
	Splenomegaly	80	21%
	Purpura	54	14%
	Mental retardation	16	4%
	Radiolucent bone disease	10	3%
	Meningo-encephalitis	7	2%

**Table 3:** Confirmed cases of CRS by country by year.

	2012	2013	2014	2015	2016	TOTAL
Burkina Faso				0	0	0
Rwanda				0	0	0
Tanzania			20	44	1	65
Zambia			1	5	0	6
Zimbabwe	14	6	2	20	6	48
Total	14	6	23	69	7	119

**Table 4:** Confirmed cases of CRS by age group. 2012 – 2016. Sentinel surveillance in 5 countries.

	0 - 28 days	1 - 5 months	6 - 11 months	1 year or more of age	Age missing	Total
Burkina Faso	0	0	0	0	0	0
Rwanda	0	0	0	0	0	0
Tanzania	5	15	44	0	1	65
Zambia	2	0	3	0	1	6
Zimbabwe	14	20	4	2	8	48
Total	21	35	51	2	10	119

**Table 5:** Clinical presentation in the 119 confirmed cases of CRS in 5 countries. 2012 – 2016.

Clinical sign		Number of cases	% cases
Group A	Congenital Heart Disease	86	72%
	Cataracts	38	32%
	Glaucoma	12	10%
	Hearing impairment	6	5%
	Pigmentary retinopathy	1	1%
Group B	Microcephaly	40	34%
	Splenomegaly	30	25%
	Jaundice	16	13%
	Purpura	14	12%
	Mental retardation	10	8%
	Meningo-encephalitis	4	3%
	Radiolucent bone disease	0	0%
